# Spinal Clear Cell Meningioma: Atypical Clinical and Radiological Manifestations

**DOI:** 10.1155/2021/9998399

**Published:** 2021-05-27

**Authors:** Mohammad Nasser Alsadiq, Zainab Shaker Albarbari, Fatimah Alshakhs, Muath Ali Alduayji, Shaymaa Al-Umran, Abdulrahman Alenzi

**Affiliations:** ^1^Department of Neurosurgery, Dammam Medical Complex, Dammam, Saudi Arabia; ^2^Department of pathology, Dammam Medical Complex, Dammam, Saudi Arabia

## Abstract

Meningioma has many subtypes with clear cell meningioma being more aggressive than other variants of meningioma and one of the rarest. We report a case of spinal clear cell meningioma that occurred in a 25-year-old lady who presented with the inability to be in the supine position. A magnetic resonance image showed an intradural mass extending from L1 to L4. Near complete excision was done. The patient had motor weakness postoperatively which improved gradually. A histopathological study showed a clear cell meningioma. In a differential diagnosis of any space-occupying lesion of the spine, clear cell meningioma should be considered though it is a rare form of meningioma due to its potential to recure. An accurate follow-up is warranted.

## 1. Introduction

Meningioma is a tumor arising from the meningothelial cells in the arachnoid villi. It is the commonest primary nonglial brain tumor which compromises around 13 to 19% of all primary intracranial neoplasm. [1]

The predominant site of involvement is the intracranial cavity (60%), while the involvement of the spinal canal constitutes 12% of all the cases of meningioma. 25% of all spinal cord tumors are spinal canal meningiomas [2–4].

In general, spinal tumors are classified to intradural and extradural tumors. Intradural tumors are further subdivided into intramedullary and extramedullary spinal tumors [5–7].

In most cases, spinal meningiomas confounded to the intradural space (90%), while 5% are extradural and the remaining 5% are both extra- and intradural [1, 8].

Spinal meningiomas are diagnosed based on the clinical manifestation and the imaging study findings. The most common clinical manifestation of spinal meningioma is a localized back pain, which is usually not radiating. The modality of choice in diagnosing spinal tumors is the magnetic resonance imaging (MRI) [9].

When a meningioma has an unfamiliar location and radiological characteristics with the absence of symptoms, the diagnosis becomes difficult. This is a case report of a spinal meningioma with atypical radiological features in a 25-year-old female.

## 2. Case Presentation

This is a case of 25-year-old Saudi lady who is a known case of epilepsy and with an intellectual disability since infancy. She presented to the clinic with a history of kyphosis for three years. She was unable to sleep in the supine position and had to sleep in the sitting position for three years. However, she was able to walk independently and had no sphincter disturbance. There was no history of loss of appetite or weight loss. She had a history of bilateral lower limb lymphedema for several years. The neurological examination at the time of presentation was normal.

**Figure 1:**
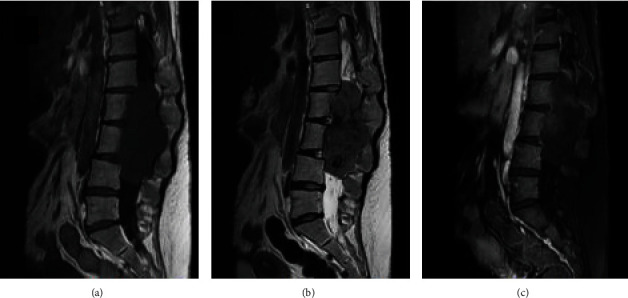
A lumbar spine MRI sagittal ((a) T1, (b) T2, and (c) T1 with contrast).

**Figure 2:**
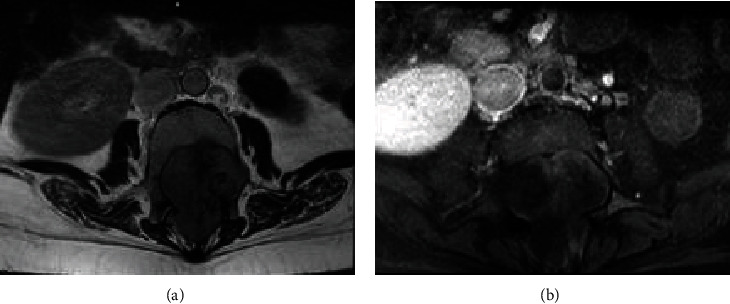
A lumbar spine MRI axial view ((a) T2 and (b) T1 with contrast).

The patient was investigated with a lumbar spine magnetic resonance imaging (MRI). It showed a large intrathecal mass extending from L1 to L4. This mass was hypointense on both T1- and T2-weighted images. The lesion was approximately 10 × 5 × 4.3 cm in diameter, and it was widening the spinal canal with scalloping of the vertebral bodies extensively seen at L3. Also, a bilateral dilatation of neural foramina was noted. This lesion was heterogeneously enhancing after injection of contrast (Figures 1 and 2).

**Figure 3:**
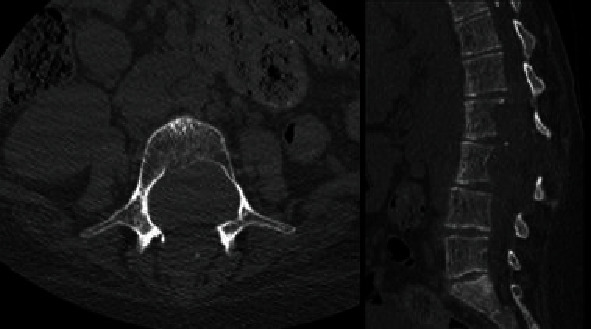
A lumbar spine CT scan: axial and sagittal views.

The patient was further investigated with a CT scan and a brain and whole spine MRI. No other lesions were seen in the spine and brain MRI. The CT scan showed an intradural spinal lesion extending from L1 to L4 widening the spinal canal and causing scalloping of vertebral bodies. The lesion is also extending to the corresponding neural foramina with widening of the foramina. A posterior extension was noted mainly at the L3 level (Figure 3).

Preoperatively, the radiologic provisional diagnosis was spinal myxopapillary ependymoma with possibility of schwannoma, neurofibroma, or meningioma (less likely).

**Figure 4:**
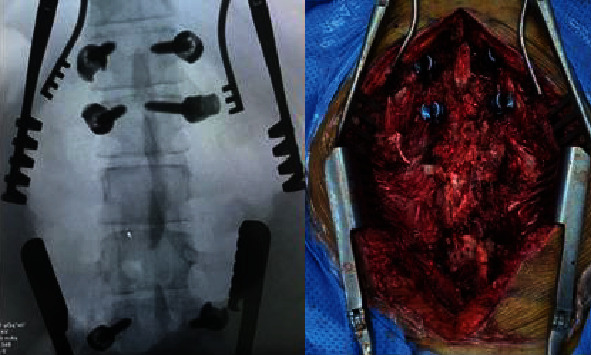
X-ray AP view and intraoperative image post-intrapedicular screw insertion and laminectomy.

The patient underwent a subtotal tumor resection and fixation. Using a posterior midline approach, a laminectomy from the lower edge of L1 to L3 was performed with fixation on T12, L1, and L4 on both sides (Figure 4).

**Figure 5:**
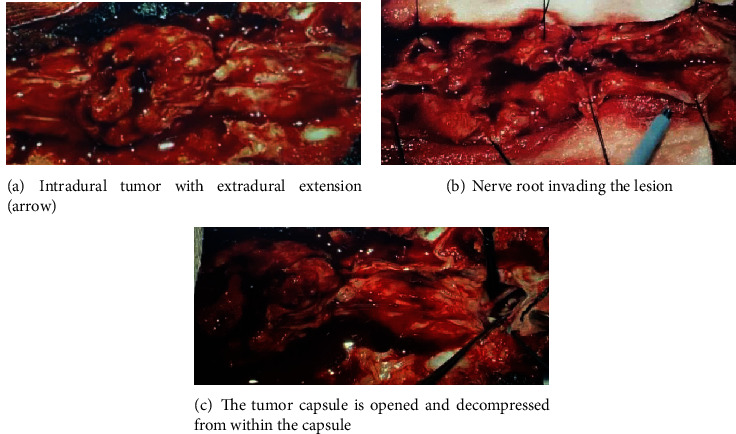
Intraoperative images for spinal tumor resection.

The mass appeared to be intradural, extending to the extradural space mainly at the L3 level. The dura was opened proximal and distal to the lesion, and it was noted that the nerve roots were scattered over the tumor surface and invading the tumor (Figures 5(a) and 5(b)). The main bulk of the spinal cord was bushed anterolateral toward the left side. Intraoperative monitoring was used throughout the operation, and tumor debulking from within the capsule was done. Almost a complete tumor resection was achieved under an operating microscope by CUSA (Figure 5(c)). Only tumor capsule was left as it was impossible to differentiate it from the thecal sac.

Postoperatively, the patient was complaining of right lower limb weakness: ankle dorsiflexion and planter flexion (1/5), knee flexion and extension (2\5), and hip flexion and extension (1/5). The power improved gradually with physiotherapy. Upon discharge, she was able to stand and walk few steps using a walking frame. Additionally, she was discharged on Foley's catheter as she was having urinary retention.

**Figure 6:**
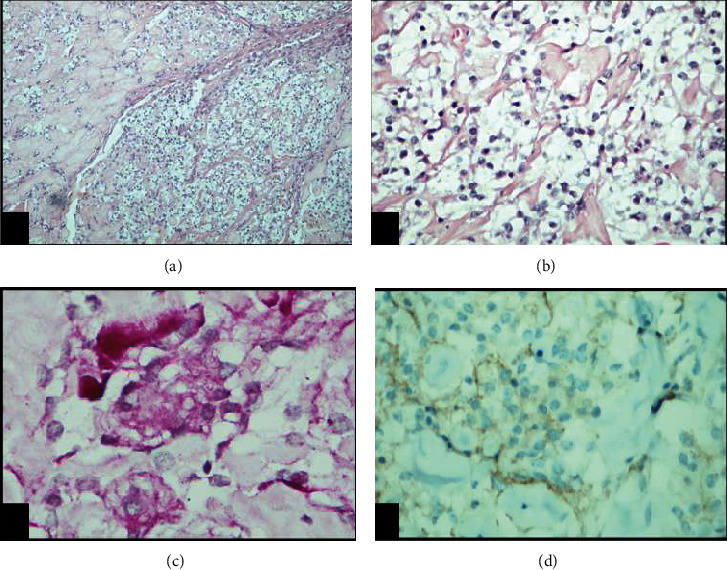
(a) Clear cell meningioma exhibiting sheets of round to polygonal cells arranged in a haphazard manner alternating with areas with thick bulky interstitial collagenization (H&E, 100x). (b) High magnification image shows lesional cells with clear cytoplasm (H&E, 400x). (c) The cytoplasm contains glycogen demonstrated by periodic acid-Schiff (PAS; 400x). (d) Weak membranous immunoreactivity for epithelial membrane antigen (IHC, 400x).

Microscopically, the tumor characterized rounded cells with a moderate amount of clear cytoplasm in a patternless arrangement and extensive perivascular and interstitial collagenization; whorl formation was very vague and focal. In addition, there are areas showing angiomatous-like features (Figures 6(a) and 6(b)). The cells demonstrated periodic acid-Schiff-positive, diastase-sensitive intracytoplasmic glycogen granules (Figure 6(c)). Immunostains show positive EMA (Figure 6(d)), S100 (rare cells), and CD99 (weak), but negative chromogranin, CD10, CAM5.2, inhibin-A, GFAP, S100, pankeratin, and synaptophysin staining. CD34 and CD31 decorate the collagenized blood vessels. The MIB-labeling index was 2%. The final pathological diagnosis was clear cell meningioma (WHO Grade II).

## 3. Discussion

Spinal meningiomas account for 12% of all meningiomas. Most of them are confined within the intradural space; having an extradural extension is uncommon. Spinal meningioma accounts for 25% of all primary spinal cord tumors [10], with females being the predominant patients accounting for approximately 80% [11]. Meningiomas located in the lumbar spine are the least common site of involvement (4%), as opposed to thoracic (80%) and the cervical spine (16%) [2]. In all the studies concerning the spinal meningioma, the thoracic region was reported as the most common sit of involvement in a literature review except for two studies, where the common site was the lumbar region [12, 13].

Clinically, most of the cases presented with motor and sensory symptoms. The most commonly reported symptom was radicular, or funicular back pain (75%), followed by limb weakness (33.3%), hypoesthesia, paresthesia (15.5%), and sphincter dysfunction (10.7%) [13–15]; conversely, our patient was asymptomatic with no motor weakness, sensory changes, or any neurological deficit. However, she was walking while keeping her trunk in a flexed position with the inability to lie in the supine position. In a study analyzing 194 patients with spinal meningioma, a total of 26 patients were reported to be asymptomatic [16, 17].

Radiologically, the MRI showed a large lobulated intrathecal lumbar expansile hypointense mass in both T1 and T2 with heterogeneous enhancement extending between L1 and L4 levels extending to corresponding neural foramina with a significant widening and attached to conus medullaris and the cauda equina. Unlike our case, other reported cases showed similar signal intensity to the spinal cord on T1- and T2-weighted images, with homogeneous strong contrast enhancement [2, 18]. Dural tail sign was found in most cases of extradural en plaque lesion suggesting the dura-based origin [18]. Nevertheless, our patient did not present any signs that clarify the dura origin of the tumor. It was challenging to radiologically diagnose the lesion as a meningioma since it could easily be mistaken with spinal myxopapillary ependymoma neurofibroma or schwannoma.

The histological diagnosis of clear cell meningioma is challenging as it lacks cellular whirling and other meningothelial features identified in typical meningiomas [19]. In addition, the extensive stromal and perivascular collagen is considered degenerative in nature and reflects the longstanding process, as in our case, it can obscure the cellular features adding to the diagnostic challenge of this tumor. The main histological differential diagnoses include other meningioma variants such as microcytic or angiomatous meningioma; however, PAS cytoplasmic reactivity and the round cell morphology highlighted by EMA are seen in clear cell meningioma but not in the other variants [20]. In addition, obscuring the cellular features by collagen adds to the challenge in diagnosis. Other differential diagnoses such as clear cell ependymoma, hemangioblastoma, and metastatic carcinoma can be ruled out by the negative glial fibrillary acidic protein, vascular markers, and keratins (including CAM2.5), respectively [21, 22]. Although the MIB-labeling index was 2%, data show no association between the MIB-labeling index and recurrence or outcome of these tumors. Higher MIB labelling has been described in recurrences [21].

## 4. Conclusion

We describe here a rare case of spinal intradural extramedullary meningioma with extradural extensions at the lumbar level with atypical presentation and radiological characteristics. However, meningiomas should be considered a differential diagnosis in a slow growing lesion as they may occur in rare locations with variable imaging findings and presentation.
